# Efficacy of cinnamon supplementation on glycolipid metabolism in T2DM diabetes: A meta-analysis and systematic review

**DOI:** 10.3389/fphys.2022.960580

**Published:** 2022-11-24

**Authors:** Qian Zhou, Xingxing Lei, Shunlian Fu, Zinan Li, Yiding Chen, Cong Long, Suwen Li, Qiu Chen

**Affiliations:** Hospital of Chengdu University of Traditional Chinese Medicine, Chengdu, Sichuan, China

**Keywords:** cinnamon, lipid, glucose, diabetes mellitus, meta-analysis

## Abstract

**Background:** Cinnamon is a spice used in cooking and in large quantities as a medical complement with hypoglycemic and lipid-lowering properties. The potential pharmacological mechanisms underlying cinnamon’s anti-diabetic properties and its active ingredients have not been adequately determined. The current meta-analysis aims to systematically review the potential pharmacological mechanisms underlying the hypoglycemic and hypolipidemic efficacy of cinnamon administration and summarize clinical recommendations of cinnamon and its active ingredients.

**Method:** Relevant randomized clinical trials (RCTs) were identified through a literature search that spanned the years January 2005 to April 2022. Retrieve electronic databases including Web of Science, PubMed, Embase, Medline, and the Cochrane Library. To obtain standardized mean differences (SMDs), continuous outcomes were pooled and 95 percent confidence intervals (CIs) were provided. Categorical outcomes were aggregated to calculate relative risks (RRs) and were accompanied by 95% CIs. Heterogeneity was measured using the Cochrane Q-test and I^2^ statistics, with a *p* < 0.05 considered as substantial heterogeneity. If I^2^ was less than 50%, a fixed effect model was employed; otherwise, a random effect model was used. Subgroup analyses and sensitivity analyses were performed to identify the origins of heterogeneity. Publication bias was retrieved by means of a funnel-plot analysis and Egger’s test. The data were analyzed using revman (V.5.3) and stata (V.15) software packages.

**Results:** These 16 RCTs included a total of 1,020 patients who were followed for a duration ranging from 40 days to 4 months. According to the current meta-analysis results, glycolipid levels in diabetic individuals who received cinnamon were significantly improved as compared to those who got placebo (All *p* < 0.05). An adverse effect was only detected in one patient.

**Conclusion:** These findings imply that cinnamon has a significant influence on lipid and glucose metabolism regulation. An even more pronounced effect was observed in patients with HbA1c of 8%. The results of this study suggested that cinnamon may be utilized as hypoglycemic and lipid-lowering supplement in clinical settings with a guaranteed safety profile.**Systematic Review Registration**: [PROSPERO], identifier [CRD42022322735].

## Introduction

Diabetes mellitus (DM) and its complications have reached epidemic levels, particularly in poorer countries, posing a major threat to global health and economies (Zheng et al., 2018). By 2040, the overall population with diabetes is predicted to further reach to approximately 642 million (uncertainty interval: 521–829 million) (Ogurtsova et al., 2017). According to clinical manifestation, DM is categorized into three main types: type 1 diabetes mellitus (T1DM), type 2 diabetes mellitus (T2DM), and gestational diabetes mellitus (GDM) (Li et al., 2017). T2DM accounts for approximately 90% of diabetes mellitus cases (Bruno et al., 2005; Holman et al., 2015). T2DM is distinguished by hyperlipidemia and hyperglycemia caused by peripheral insulin resistance or impaired insulin production in the pancreas (Wang et al., 2014; Chatterjee et al., 2017). Furthermore, risk factors such as endothelial dysfunction, oxidative stress, vascular atherosclerosis, obesity, and inflammatory processes are also associated with T2DM (Petrie et al., 2018; Zheng et al., 2018). As the cornerstone of diabetes management, diet has a substantial impact on postprandial glucose and general physical health, and many edible plant species have been exploited as diabetic traditional Chinese medicines (TCMs) (Shapira, 2019). In recent years, large-scale clinical trials have demonstrated that TCM for pharmaceutical and dietary therapy has progressed in managing blood glucose and cholesterol levels, with greater effectiveness and fewer side effects (Tian et al., 2019). This is mainly attributed to the whole perspective and multiple target approaches of TCM providing distinct benefits in the management of complicated diabetes (Tong et al., 2012). Cinnamon, a spice and/or flavoring ingredient, has emerged as a promising supplement for the treatment of T2DM, obesity, and dyslipidemia (Santos and da Silva, 2018). However, the mechanisms and effectiveness of cinnamon on metabolic diseases remain perplexing because large majority of studies exhibited design limitations, such as short intervention durations, limited sample sizes, and inconsistent efficacy assessments (Gannon et al., 2015).

Several lines of evidence supported the effectiveness of cinnamon on promoting lipolysis and fatty acid oxidation to modulate diabetes status (Huang et al., 2011; Khare et al., 2016) in enhancing the catabolism of fats and protein is an effective option for maintaining glycolipid homeostasis (Babu et al., 2013). However, there are several limitations in pre-publication meta-analyses resulting in a failure to elucidate a comprehensive conclusion on the role of cinnamon in the treatment of diabetes due to high heterogeneity.

In the present meta-analysis, we explore the basic theory of cinnamon for diabetes treatment, assess recent clinical practice achievements, and data from new pharmacological studies utilizing cinnamon to systematically review the efficacy of cinnamon on glycolipid metabolism regulation in T2DM patients and identify potential anti-diabetic mechanisms. The parameters of safety were also explained. Moreover, we summarize the effective extraction ingredients of cinnamon and their distinct roles in diabetes therapy. Comprehending the action of cinnamon on diabetes could stimulate the development of novel therapeutic methodologies and potential foundations for future individualized comprehensive diabetes therapy.

## Materials and methods

The present systematic review and meta-analysis was designed and carried out in accordance with the preferred guidance of the Reporting Items for Systematic Reviews and Meta Analysis (PRISMA) 2020 (Page et al., 2021) (Supplementary Table S1), A Measurement Tool to Assess Systematic Reviews 2 (AMSTAR 2) (Shea et al., 2017) (Supplementary Table S2), and Methodological Expectations of Cochrane Intervention Reviews (MECIR) (Lefebvre et al., 2013) guidelines for conducting, reporting, and updating of systematic reviews. The protocol of this study has been registered (PROSPERO registration number CRD42022322735).

### Search strategy and study selection

A systematic search for relevant literature was conducted for all relevant randomized clinical trials (RCTs). Relevant RCTs were identified through literature search that published from January 2005 to April 2022. Retrieve electronic databases including Web of Science, PubMed, Embase, Medline, and the Cochrane Library. Search strategies included medical subject heading (MeSH) terms and scientific names of the keywords. The terms included “diabetes” for the population; “glucose and lipid” were used for exposures, and “cinnamon” was used for interventions. The search for articles was based on the scientific name of the keywords and the common name. The search strategy for PubMed is presented in Supplementary Table S3. EndNote (version X9) is used to manage the literature search results.

Clinical trials that satisfied the following criteria were included: 1) the original paper published in peer-reviewed journals; 2) RCTs with parallel group or cross-over design in humans; 3) participants in the research had a confirmed diagnosis of diabetes; 4) participants were randomly assigned to receive cinnamon, Western medicine, or placebo; 5) no limitation on the dosage or duration of treatment; 6) primary outcomes; and 7) methodological quality. Clinical trials with the following characteristics were excluded: 1) not randomized studies; 2) patients with no definite diagnosis; 3) studies that simply documented symptomatic changes in patients without a physical examination or objective laboratory measurement; and 4) the articles or non-research articles were unpublished.

### Data extraction

Two researchers worked separately to gather data from the included studies. The study’s baseline data and demographic characteristics were retrieved. The name of the first author, publication year, study group, and sample size for each group, age, total cholesterol (TC), triacylglycerol (TG), high-density lipoprotein cholesterol (HDLc), low-density lipoprotein cholesterol (LDLc), homeostatic model assessment of insulin resistance (HOMA-IR), body mass index (BMI), glycated hemoglobin A1c (HbA1c), insulin, and fasting plasma glucose (FPG) level were all included in the baseline information. Where necessary, study authors were contacted for additional information. The effective extraction ingredients of cinnamon and their distinct roles and side effects in diabetes therapy in high-quality trials were also summarized.

### Literature quality and GRADE assessment

The literature quality and bias of all eligible studies were assessed using the Cochrane Collaboration Risk of Bias Tool and standard Excel forms. We evaluated the quality of studies using the following seven criteria: 1) Random sequence generation, 2) allocation concealment, 3) blinding of participants and personnel, 4) blinding of outcome assessment, 5) incomplete outcome data, 6) selective reporting, and 7) other possible risk biases. The assessment of evidence quality was performed according to the Grading of Recommendations Assessment, Development, and Evaluation (GRADE) process (Morgenthaler et al., 2016).

Study screening and selecting, data extracting, quality evaluation of eligible studies, and statistical analysis were all completed independently by two researchers (ZL and YC). Any ambiguity or inconsistency in this course was resolved *via* discussion and the participation a third researcher (QZ).

### Statistical analysis

Revman (V.5.3) and stata (V.15) software packages were used to examine the data. To obtain standardized mean differences (SMDs), continuous outcomes were pooled, and 95% confidence intervals (CIs) were provided. Categorical outcomes were aggregated to calculate relative risks (RRs), accompanied by 95% CIs. Heterogeneity was measured using the Cochrane Q-test and I^2^ statistics, with a *p* < 0.05 considered as substantial heterogeneity (Melsen et al., 2014). Subgroup analyses were performed to evaluate the probable sources of heterogeneity. Where necessary, individual studies that may have influenced the results were removed where appropriate, using sensitivity analysis. Where I^2^ was less than 50%, a fixed effect model was employed; otherwise, a random effect model was used. A funnel-plot analysis and Egger’s test (Egger et al., 1997) (>10 studies) with a *p* < 0.05 were used to indicated potential publication bias. When there was a publication bias, the pooled estimates were corrected using “trim and fill” methods (Peters et al., 2007).

## Results

### Literature screening results

**FIGURE 1:**
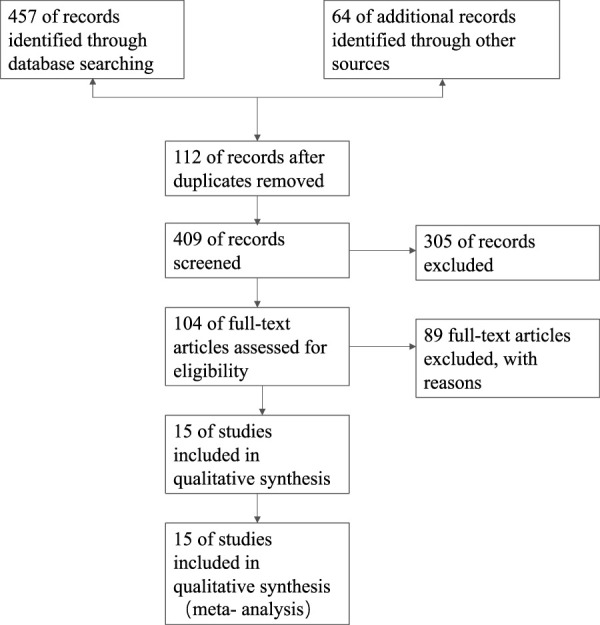
Flowchart of literature search.

The literature search yielded 521 titles, from which 409 abstracts were retrieved, as shown in Figure 1. A total of 104 abstracts were deemed possibly relevant, and the full-text articles were retrieved. Finally, 15 full-text articles (Mang et al., 2006; Vanschoonbeek et al., 2006; Blevins et al., 2007; Crawford, 2009; Akilen et al., 2010; Wainstein et al., 2011; Lu et al., 2012; Vafa et al., 2012; Hasanzade et al., 2013; Azimi et al., 2014; Mirfeizi et al., 2016; Talaei et al., 2017; Zare et al., 2019; Mirmiranpour et al., 2020; Lira Neto et al., 2021) were included, which encompassed the use of cinnamon powder or aqueous cinnamon extracts. A total of 1,020 patients were included in these 15 randomized controlled trials, with follow-up ranging from 40 days to 4 months. In one experiment, multiple dose levels were employed simultaneously (Lu et al., 2012). The number of cases included in these articles varied between 4 and 1,572. In the included 15 RCTs, 14 administered cinnamon powder and one administered aqueous cinnamon extracts with doses ranging from 1 to 6 g per day based on their previous diet, physical activity, and medicines. The control groups received conventional therapy as before. The baseline characteristics of the study participants are given in Table 1.

**TABLE 1:** Characteristics of included studies.

Author	Publication year	Population/Country	Sample size (control/intervention)		Daily dose	Main characteristics of medications	Duration	Age (y) (control/intervention)	
			Control	Intervention				Control	Intervention
Vanschoonbeek et al., 2006	2006	Netherlands	13	12	1.5 g/d	Oral antidiabetic	6 weeks	64 ± 2	62 ± 2
Mang et al., 2006	2006	Germany	32	33	3 g/d	Oral antidiabetic	16 weeks	63·7 ± 7·17	62·8 ± 8·37
Blevins et al., 2007	2007	United States	28	29	1 g/d	Oral antidiabetic and hypolipidemia	12 weeks		
Crawford, 2009	2009	United States	54	55	1 g/d	Oral antidiabetic and insulin	12 weeks	59.9 ± 9.2	60.5 ± 10.7
Akilen et al., 2010	2010	United Kingdom	28	30	2 g/d	Oral antidiabetic	12 weeks	54.43 ± 12.53	54.90 ± 10.14
Wainstein et al., 2011	2011	Israel	30	29	1.2 g/d	Metformin and/or sulfonylurea	12 weeks	64.4 ± 15.4	61.7 ± 6.3
Vafa et al., 2012	2012	Iran	18	19	3 g/d	Metformin and gliclazide	8 weeks	55.67 ± 7.98	54.11 ± 10.37
Lu et al., 2012	2012	China	20	23	4.8 g/d	Gliclazide	12 weeks	60 ± 5.9	62.4 ± 7.9
Lu et al., 2012	2012	China	20	23	14.4 g/d	Gliclazide	12 weeks	60 ± 5.9	58.9 ± 6.4
Hasanzade et al., 2013	2013	Iran	35	35	1 g/d	Oral antidiabetic	8 weeks	54.7 ± 8.1	53.7 ± 9.7
Azimi et al., 2014	2014	Iran	39	40	3 g/d	Metformin and glibenclamide	8 weeks	53.64 ± 1.3	54.15 ± 1.0
Mirfeizi et al., 2016	2016	Iran	30	30	1 g/d	Sulfonylurea, biguanides, and/or thiazolidines	12 weeks	54 ± 12	55 ± 10
Talaei et al., 2017	2017	Iran	19	20	3 g/d	Metformin and insulin	8 weeks	56.26 ± 9.46	58.90 ± 7.93
Zare et al., 2019	2019	Iran	69	69	1 g/d	Oral hypoglycemic agents	12 weeks	53.2 ± 8.5	52.1 ± 9.7
Mirmiranpour et al., 2020	2020	Iran	27	28	0.5 g/d	Oral antidiabetic	12 weeks	58.2 ± 11	58.8 ± 12.8
Lira Neto et al., 2021	2021	United States	69	71	3 g/d	Oral antidiabetic	12 weeks	60.8 ± 10.8	61.7 ± 11.7

### Quantitative data analysis

**FIGURE 2:**
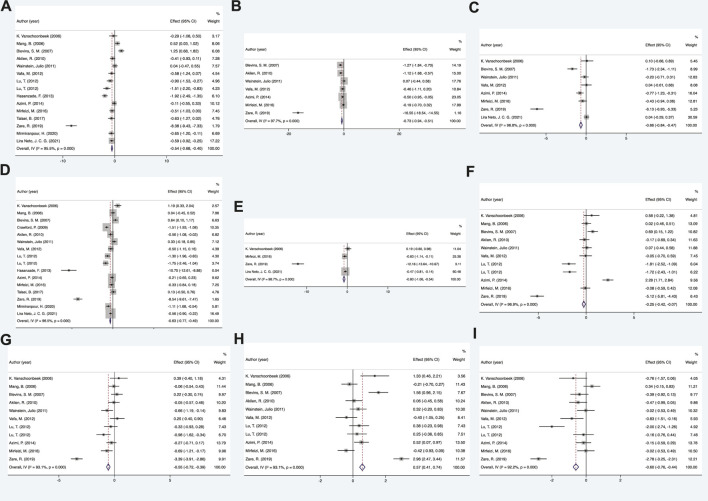
Mean difference in the changes in glycolipid metabolism indexes. **(A)** FPG, **(B)** BMI, **(C)** insulin, **(D)** HbA1c, **(E)** HOMI-IR, **(F)** TC, **(G)** LDL-c, **(H)** HDL-c, and **(I)** TG. FPG, fasting plasma glucose; BMI, body mass index; HbA1c, glycated hemoglobin A1c; HOMA-IR, homeostatic model assessment of insulin resistance; TC, total cholesterol; LDL-c, low-density lipoprotein cholesterol; HDL-c, high-density lipoprotein cholesterol; TG, triacylglycerol.

The analysis demonstrated that as a Multitarget-TCM agent, cinnamon showed significant effects in regulating glucolipid metabolism in diabetic patients compared to placebo (all *p* < 0.05). The comprehensive results are shown in Figure 2 and Supplementary Table S4.

#### Glucose metabolism

A total of 15 RCTs were included to examine the difference in fasting plasma glucose (FPG) between the cinnamon and placebo groups. Statistical analysis results of cinnamon interventions on glucose metabolism for diabetes are summarized in Supplementary Table S4. The random-effects model findings from the meta-analysis showed that the changes in FPG of the cinnamon group had statistical significance when compared with control group, showing that cinnamon can effectively lower FPG levels in patients (SMD = −0.54, 95% CI: −0.68 to −0.4, *p* < 0.05). Regarding other indicators of glucose metabolism, the results showed that diabetes patients who received cinnamon compared with placebo was found to be beneficial in lowering HbA1c (SMD = −0.63, 95% CI: −0.77 to −0.49, *p* < 0.05), insulin (SMD = −0.66, 95% CI: −0.84 to −0.47, *p* < 0.05) and HOMA-IR (SMD = −0.80, 95% CI: −1.06 to −0.54, *p* < 0.05).

#### Lipid metabolism

Ultimately, 11 RCTs were included to compare the changing in lipid metabolism between the cinnamon and control groups. As a 6–12-week follow-up, the lipid metabolism was significantly improved in cinnamon groups. More data details are summarized in Supplementary Table S4. For total cholesterol (TC), the results of present meta-analysis indicated that there was a significant reduction after cinnamon treatment (SMD = −0.25, 95% CI: −0.42 to −0.07, *p* < 0.05). Similar results were obtained for other indicators of lipid metabolism, such as low-density-lipoprotein cholesterol (LDL-c) (SMD = −0.55, 95% CI: −0.72 to −0.39, *p* < 0.05) and triacylglycerol (SMD = −0.60, 95% CI: −0.76 to −0.44, *p* < 0.05). The HDL cholesterol level was substantially increased following cinnamon administration compared with the placebo group (SMD = 0.57, 95% CI: 0.41 to 0.74, *p* < 0.05).

#### Other anthropometric indicators

Among anthropometric measures, BMI was the most focused and representative characteristic. Our meta-analysis indicated that BMI was significantly improved after cinnamon administration compared with the placebo group (SMD = −0.75, 95% CI: −0.94 to −0.51, *p* < 0.05).

### Subgroup and sensitivity analyses

We conducted subgroup analyses and sensitivity analyses to identify the sources of heterogeneity by varying multiple parameters simultaneously. Pre-planned subgroup analyses for some parameters were performed for the primary outcomes (Triacylglycerol, HDL cholesterol, LDL cholesterol, total cholesterol, HbA1c, and FBG) with a representation number of included trials. Details of all subgroup analyses scenarios are provided in Table 2. Due to the diversity of interventions, subgroup analyses of treatment duration and dose were required due to the diversity of interventions. Moreover, for evaluating long-term glycemic control, hemoglobin A1c levels would have been more appropriate (Derr et al., 2009). Then, we performed subgroup analysis according to different dosages, duration, medications, and hemoglobin A1c levels. The subgroups of doses were separated into two categories: low-dose group (<3 g/day) and the high-dose group (≥3 g/day). The duration was divided into two subgroups: short-term subgroup (<12 weeks) and long-term subgroup (≥12 weeks). The HbA1c levels were stratified into high-level (HbA1c ≥ 8%) and low-level (HbA1c < 8%) subgroups. More comprehensive analysis on subgroups with a representative number of trials yielded some intriguing results. For HbA1c, the results were completely opposite for the high- and low-level subgroups assessed. When the HbA1c < 8%, no statistical significance was observed within each subgroup. On the contrary, the subgroup analyses presented significantly improved for these primary outcomes in high-level (HbA1c ≥ 8%) subgroups. Similar statistically significant treatment results were observed within each subgroup, except for the subgroup of HDL cholesterol and total cholesterol among the high-dose subgroups and LDL cholesterol and HbA1c among the short-term subgroups. After subgroup analysis, we discovered that heterogeneity was remained considerably high when compared to previous studies. We therefore performed further sensitivity analyses for each end point by excluding individual studies. The results of the sensitivity-pooled SMD on the bulk of the outcomes indicated that all exclusions had no effect on the prior analyses results.

**TABLE 2:** Summary of subgroup analysis with random effects SMD (95% CI). ^*^Statistically significant variables at *p*-value <0.05.

Primary outcome	HbA1c		Dose		Duration	
	HbA1c<8%	HbA1c ≥ 8%	<3 g/d	≥3 g/d	<12 weeks	≥12 weeks
Triacylglycerol	−0.16 (−0.39, 0.06)	−1.07 (−1.31, −0.83)	−0.81 (−1.03, −0.59)	−0.33 (−0.58, −0.08)	−0.42 (−0.76, −0.09)	−0.66 (−0.84, −0.47)
	*p* = 0.158	*p* < 0.05	*p* < 0.05	*p* < 0.05	*p* < 0.05	*p* < 0.05
HDL cholesterol	0.21 (−0.02, 0.45)	0.93 (0.69, 1.16)	0.94 (0.72, 1.160)	0.14 (−0.1.0.39)	0.39 (0.05, 0.73)	0.63 (0.44, 0.81)
	*p* = 0.074	*p* < 0.05	*p* < 0.05	*p* = 0.244	*p* < 0.05	*p* < 0.05
LDL cholesterol	−0.16 (−0.39, 0.06)	−0.97 (−1.21, −0.74)	−0.80 (−1.03, −0.58)	−0.26 (−0.5, −0.01)	−0.02 (−0.35, 0.32)	−0.73 (−0.92, −0.54)
	*p* = 0.16	*p* < 0.05	*p* < 0.05	*p* < 0.05	*p* = 0.923	*p* < 0.05
Total cholesterol	0.17 (−0.06.0.39)	−0.87 (−1.15, −0.59)	−0.043 (−0.66, −0.20)	0.00 (−0.27, 0.27)	1.11 (0.73, 1.48)	−0.63 (−0.83, −0.43)
	*p* = 0.153	*p* < 0.05	*p* < 0.05	*p* = 0.995	*p* < 0.05	*p* < 0.05
HbA1c	−0.21 (−0.43, 0.01)	−0.91 (−1.09, −0.74)	−0.79 (−0.99, −0.59)	−0.48 (−0.67, −0.29)	−0.29 (−0.58, 0.00)	−0.73 (−0.88, −0.57)
	*p* = 0.059	*p* < 0.05	*p* < 0.05	*p* < 0.05	*p* = 0.053	*p* < 0.05
Fpg	−0.18 (−0.40, 0.04)	−0.80 (−0.98, −0.61)	−0.67 (−0.88, −0.46)	−0.44 (−0.63, −0.25)	−0.68 (−0.94, −0.41)	−0.49 (−0.66, −0.32)
	*p* = 0.1	*p* < 0.05	*p* < 0.05	*p* < 0.05	*p* < 0.05	*p* < 0.05

### Publication bias and quality assessment

**FIGURE 3:**
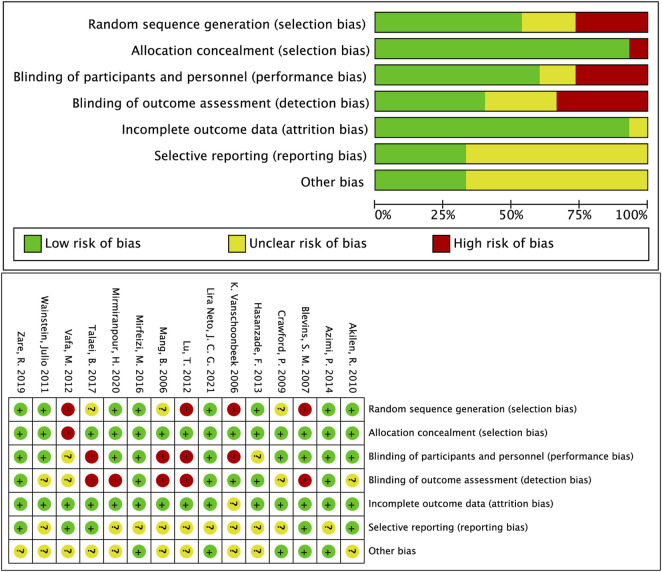
Overall summary of risk of bias in the included studies. +: low risk of bias; −: high risk of bias; ?: unclear risk of bias.

Judgments about each risk-of-bias item for all eligible studies made using the Cochrane Collaboration tool (Higgins et al., 2011) to assess the methodological quality and bias. The detailed results of each item are presented in Figure 3. The GRADE approach evidence certainty and summary of findings of the clinical important outcomes in Table 3. A publication bias funnel plot was used to visually assess the presence of potential publication bias. Symmetrical dispersion points (Supplementary Figure S1) and the Egger test suggested no evident publication bias was identified for cinnamon on FPG (*p* = 0.051), TG (*p* = 0.684), HDL-c (*p* = 0.967), LDL-C (*p* = 0.717), and TC (*p* = 0.178). Moreover, there was observable publication bias among the included studies for HbA1c (Egger *p* = 0.025). After the exclusion of Hasanzade et al. (2013) and Zare et al. (2019), we found a significant reduction in publication bias (Egger *p* = 0.617). The overall major conclusions did not alter following the elimination of studies having a high risk of bias in the cumulative analysis.

**TABLE 3:** Grading of Recommendations Assessment, Development, and Evaluation (GRADE) approach evidence certainty and summary of findings of the clinical important outcomes.

**Summary of findings:**						
**Cinnamon compared to control for type 2 diabetes mellitus**						
**Patient or population**: Type 2 diabetes mellitus						
**Setting**:						
**Intervention**: Cinnamon						
**Comparison**: Control						

Outcomes	Anticipated absolute effects^e^ (95% CI)		Relative effect (95% CI)	No. of participants (studies)	Certainty of the evidence (GRADE)	Comment
	Risk with control	Risk with Cinnamon				
Fasting plasma glucose follow-up: range 6 weeks to 16 weeks	**-**	SMD **0.54 SD lower** (0.68 lower to 0.4 lower)	-	968 (15 RCTs)	⊕⊕○○ Low^a^ ^,^ ^b^	No explanation was provided
Body mass index follow-up: range 8 weeks to 12 weeks	-	MD **0.73 SD lower** (0.94 lower to 0.51 lower)	-	488 (7 RCTs)	⊕⊕○○ Low^c^ ^,^ ^d^	No explanation was provided
Hemoglobin A1c follow-up: range 6 weeks to 16 weeks	-	SMD **0.63 SD lower** (0.77 lower to 0.49 lower)	-	1077 (16 RCTs)	⊕⊕⊕○ Moderate^a^ ^,^ ^b^	No explanation was provided
Insulin follow-up: range 6 weeks to 12 weeks	-	SMD **0.66 SD lower** (0.84 lower to 0.47 lower)	-	595 (8 RCTs)	⊕⊕○○ Low^c^ ^,^ ^d^	No explanation was provided
Homeostatic model assessment of insulin resistance follow-up: range 6 weeks to 12 weeks	-	SMD **0.8 SD lower** (1.06 lower to 0.54 lower)	-	363 (4 RCTs)	⊕⊕○○ Low^c^ ^,^ ^d^	No explanation was provided
Triacylglycerol follow-up: range 6 weeks to 16 weeks	-	SMD **0.6 SD lower** (0.76 lower to 0.44 lower)	-	665 (11 RCTs)	⊕⊕⊕○ Moderate^a^	No explanation was provided
Total cholesterol follow-up: range 6 weeks to 16 weeks	-	SMD **0.25 SD lower** (0.42 lower to 0.07 lower)	-	664 (11 RCTs)	⊕⊕⊕○ Moderate^a^	No explanation was provided
Low-density lipoprotein cholesterol follow-up: range 6 weeks to 16 weeks	-	SMD **0.55 SD lower** (0.72 lower to 0.39 lower)	-	664 (11 RCTs)	⊕⊕⊕○ Moderate^a^	No explanation was provided
High-density lipoprotein cholesterol follow-up: range 6 weeks to 16 weeks	-	SMD **0.57 SD higher** (0.41 higher to 0.74 higher)	-	664 (11 RCTs)	⊕⊕⊕○ Moderate^a^	No explanation was provided

CI: confidence interval; MD: mean difference; SMD: standardized mean difference. GRADE Working Group grades of evidence. High certainty: we are very confident that the true effect lies close to that of the estimate of the effect. Moderate certainty: we are moderately confident in the effect estimate: the true effect is likely to be close to the estimate of the effect, but there is a possibility that it is substantially different. Low certainty: our confidence in the effect estimate is limited: the true effect may be substantially different from the estimate of the effect. Very low certainty: we have very little confidence in the effect estimate: the true effect is likely to be substantially different from the estimate of the effect.

^a^ Differences in Vanschoonbeek et al. may be attributed to the inclusion of postmenopausal type 2 diabetes women; those of Akilen et al. and Zare et al. could be attributed to the fact that they studied only patients with poorly controlled type 2 diabetes.

^b^ Subjects of Vanschoonbeek et al. and Hasanzade et al.’s studies had time (6 weeks and 8 weeks) cinnamon dosage (1.5 g/d and 1 g/d) limitations.

^c^ There are obvious weaknesses in a few included studies.

^d^ The number of articles included is less than 10.

^e^ The risk in the intervention group (and its 95% confidence interval) is based on the assumed risk in the comparison group and the relative effect of the intervention (and its 95% CI).

## Discussion

In this meta-analysis, blood glycolipid levels were improved dramatically in diabetes patients who received cinnamon instead of a placebo (all *p* < 0.05, Figure 2). After subgroup analyses for certain primary outcomes, several important summary findings emerged. For different levels of HbA1c in particular, the results were not the same as those identified in the previously mentioned analyses. Therefore, we speculated that it would be more effective for patients with relatively severe diabetes (HbA1c ≥ 8%) to use cinnamon on regulating glycolipid metabolism. The conclusion of meta-analysis has certain limitation with the high heterogeneity among the included studies due to conflicted results about the efficacy of cinnamon in previous trials. Discrepancies among existing studies might be linked to differences in inclusion criteria, inadequate sample size, and characteristics existing in research design, such as study demographics, statistical analyses, concomitant conditions, and the dose and formulation of cinnamon used. We addressed a number of these potential sources of heterogeneity by performing sensitivity analyses to support the robustness of our preliminary analysis results. A small number of included studies had significant deficiencies. Specifically, pediatric, adult, and geriatric patients were included by Crawford (2009) with standard off-the-shelf cinnamon capsules of varying purity and active compounds. This lack of placebo may cause serious bias. The differences in patient outcome in the study by Vanschoonbeek et al. (2006) may be attributed to the inclusion of postmenopausal women with T2DM. Subjects in Vanschoonbeek et al. (2006) and Hasanzade et al. (2013) had time (6 weeks and 8 weeks) cinnamon dosage (1.5 g/d and 1 g/d) limitations. As the lifespan of an erythrocyte is around 120 days (Shemin and Rittenberg, 1946), HbA1c levels reflect average plasma glucose levels over a minimum of 2–3 months (Nathan et al., 2007). Akilen et al. (2010) and Zare et al. (2019) saw greater treatment effects. This might be attributed to the fact that only patients with poorly controlled T2DM were studied. Based on this, these five studies could be the major sources of heterogeneity.

The use of TCM for treatment of diabetes, alone or in combination with other therapies, has increased due to the negative effects associated with oral hypoglycemic medications and insulin used to treat DMs (Ahmadi et al., 2021). The holistic approach of TCM to diabetic therapy based on symptom distinction, metabolic balance, and varied administration routes may complement the treatment in Western medicine (Tong et al., 2012). Many cinnamon-based medications are readily accessible on the market, and diabetes patients utilize them on a regularly basis, sometimes preferring over allopathic therapy. This interest has grown exponentially in developing TCM therapeutic agents and their bioactive compounds as an alternate therapy for DM (William et al., 2019). Studies have shown that cinnamon has many beneficial health effects, including hypolipidemic, hypotensive, anti-inflammatory, antimicrobial, hypoglycemic, and neuroprotective activities, some of which are closely related to the prognosis and development of DM (Kwon et al., 2009; Nabavi et al., 2015; Mahmoodnia et al., 2017; Shang et al., 2021). The most common essential oil constituents of common identified in the literature include, cinnamaldehyde (Subash Babu et al., 2007), eugenol (Singh et al., 2016), beta-caryophyllene (Stevens and Allred, 2022), and gallic acid (Fernandes and Salgado, 2016), which may have major biological effects. In the following, we elaborate on these results as follows and discuss the mechanisms of cinnamon bioactive compounds.

### Pharmacological effects of cinnamon ingredients

#### Hypoglycemic and lipid-lowering mechanisms

The majority of studies (Santos and da Silva, 2018) have shown that cinnamon in dosages of 1–6 g/day resulted in a reduction in LDLc, TG, and TC in T2DM individuals, confirming the protective function of cinnamon and cinnamon extracts at various stages of diabetes, which is similar to the current meta-analysis results. Animal investigations, both *in vitro* and *in vivo*, have found that cinnamon is an insulin sensitizer (Talpur et al., 2005). However, the mechanisms and effectiveness of cinnamon on diabetes have not been fully clarified (Medagama, 2015). Because cinnamaldehyde is the principal active ingredient of cinnamon, studies have shown that cinnamaldehyde appears to be more effective than metformin in lowering blood glucose concentrations (Subash Babu et al., 2007; Sharma et al., 2020). There is evidence that the potential hypoglycemic effects of cinnamaldehyde through the role in increasing hepatic glycogen synthesis and inhibiting of gluconeogenesis (Anand et al., 2010). It also boosts the expression of receptor proteins involved in glucose transport, insulin signaling, and dyslipidemia regulation (Subash Babu et al., 2007; Sharma et al., 2020). Santos and da Silva (2018) indicated six pathways by which cinnamon enhances serum parameters and lipid reduction. Based on the aforementioned summary, this review comprehensively introduced and summarized the mechanisms in more detailly in Supplementary Table S4. We discovered that different components of cinnamon extract had distinct effects on diabetes, as indicated in Table 4, and the primary bioactivity of cinnamon is derived from interaction between phytochemicals and themselves.

**TABLE 4:** Main components present in cinnamon and their mechanisms of action.

Cinnamon oil	Structure	Molecular formula	Effect	Mechanism
Cinnamaldehyde	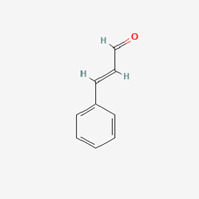	C_9_H_8_O https://pubchem.ncbi.nlm.nih.gov/compound/Cinnamaldehyde	Anti- lipidemic and anti-hyperglycemic	1. Improvement in enzyme activity, including those directly engaged in glucose metabolism and those involved in excretion (Stevens and Allred, 2022)
Eugenol	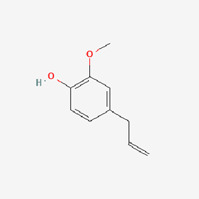	C_10_H_12_O_2_ https://pubchem.ncbi.nlm.nih.gov/compound/Eugenol	Anti-hyperglycemic, antioxidant, antibacterial, and anti-inflammatory	2. Altering ghrelin secretion and their effects on food intake and gastric emptying (Anand et al., 2010)
Beta-caryophyllene	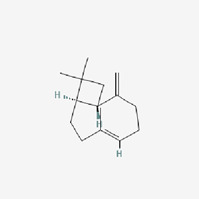	C_15_H_24_ https://pubchem.ncbi.nlm.nih.gov/compound/beta-Caryophyllene	Anti-hyperglycemic antioxidant, anti-inflammatory, and anti-lipidemic	3. Providing sympathetic actions; increased noradrenaline and thermogenic action (Saito et al., 2015)
Gallic acid	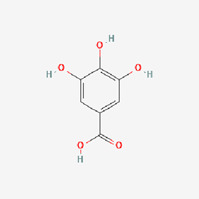	C_7_H_6_O_5_ https://pubchem.ncbi.nlm.nih.gov/compound/Gallic-acid	Antioxidant, antimicrobial, anti-inflammatory, and anticancer (Fernandes and Salgado, 2016)	4. Boosting insulin sensitivity through increasing insulin receptor mRNA and protein expression (IR) (Mani et al., 2021)
				5. Enhancing the expression of proteins involved in glucose transport (Stevens and Allred, 2022)
				6. Acting as a-glycosidase inhibitors (Singh et al., 2016)
				7. Protecting β-cells by alleviating hyperglycemia (Basha and Sankaranarayanan, 2016)

#### Other potential mechanisms

In addition to those previously mentioned, cinnamon possesses antibacterial and antioxidant qualities, and its application has been recommended individually or as a supplement in the treatment of cancers such as promyelocytic leukemia (Jayaprakasha and Rao, 2011; Assadollahi et al., 2013). Research has shown that cinnamon and cinnamon extracts are effective in alleviating diabetes-induced oxidative stress (OS), which plays an important role in the development of diabetic complications. (Niazmand et al., 2021). Finally, cinnamon is frequently used to cure inflammatory and menstrual diseases in China (Ghosh et al., 2015).

### Evaluation of adverse events

None of the included clinical trials except the two performed by Mirfeizi et al. (2016) and Crawford (2009) reported any side effects. In the two studies, one patient in each treatment group reported developing skin allergy characterized by a sudden rash that resolved after discontinuing cinnamon. Only in one subject in each study, the side effect was observed and no further adverse effects were identified. The Food and Drug Administration (FDA) of the United States has classified cinnamon as a substance generally recognized as safe to consume (https://www.fda.gov/). An umbrella review of known research on the safety of cinnamon concluded that cinnamon did not generate clear increased harmful effects when used on a large scale (Gu et al., 2021). Moreover, systematic review summarized gastrointestinal events and skin rash were the most common side-effects of cinnamon, regardless of their disease background (Hajimonfarednejad et al., 2019). Almost all cases were asymptomatic after the elimination of cinnamon. Several compounds within cinnamon have been identified as a possible source of the potential sensitization process. For most TCMs, the absence of scientific and clinical data confirming their efficacy and safety is the main obstacle prohibiting their usage in allopathic medicine for the treatment of diabetes. More clinical studies of cinnamon are needed to assess their pharmacological and toxicological utility, as well as the development of animal models for toxicity and safety testing.

Many pharmaceutical products are created from prototypic compounds found in medicinal plants. To manufacture efficacious medications, it is also necessary to identify the active components in these plant extracts. Available data suggest that cinnamon could act as an alternate therapy for diabetes by increasing insulin sensitivity and secretion; controlling glucose-related enzyme activity; regulating hepatic glucose metabolism, adipose tissue, and muscle; relieving oxidative stress and inflammation; and inhibiting the development of diabetes complications. The results of the meta-analysis suggested that cinnamon may be utilized as an adjuvant medicine in the clinic field in the future with guaranteed safety.

## Limitations

There were several limitations to this meta‐analysis. The diverse nature of the selected trials was difficult to take into account. For example, cinnamon dosage, duration of studies, lack of verification of double blinding in some RCTs, differences in demographics and clinical characteristics of participants, and background glucose-lowering treatment were not consistent, which could bring many difficulties in drawing useful conclusions from a combined analysis. However, considering the available results and information from the meta‐analysis and systematic review, cinnamon supplementation could be as a viable addition to conventional diabetes management for T2DM patients with HbA1c ≥ 8%.

## Conclusion

This updated meta-analysis of a series of participants was designed to systematically assess the effects of cinnamon on glucose and lipid levels in patients with T2DM. Cinnamon supplementation was observed to exert a favorable impact on metabolic (especially glucose, BMI, and lipids) abnormalities (all *p* < 0.05). The effect was more significant when HbA1c ≥ 8%. The findings revealed a beneficial medication for the progression of hypoglycemic and lipid lowering with cinnamon and cinnamon extract, implicating the prospective as therapeutic agents that might ameliorate hyperglycemia in diabetes, which could inspire the development of new treatment approaches, providing a novel methodology for individualized comprehensive diabetes therapy. Although numerous plants possess hypoglycemic properties and are utilized in traditional folk medicine, only a few have been scientifically and medically evaluated to assess their efficacy. We anticipate that our current work will serve as a stimulus for TCM undertakings. To shed more light on the therapeutic effects of cinnamon on diabetes patients, further investigations of the matter with longer durations, varied doses, and different age ranges are necessary.

## Data Availability

The original contributions presented in the study are included in the article/Supplementary Material; further inquiries can be directed to the corresponding author.
