# Supplementary material for: Efficacy of cinnamon supplementation on glycolipid metabolism in T2DM diabetes: A meta-analysis and systematic review
Source: Front Physiol. 2022 Nov 24;13:960580. doi: 10.3389/fphys.2022.960580 (PMC9731104; doi:10.3389/fphys.2022.960580)
Supplement: Supplementary file 2 [file DataSheet1.docx]

Supplementary figure 1. Funnel plot for publication bias analysis of the selected studies. **(A)** FPG, (B) HbA1c, (C) TC, (D) LDL-c, (E) HDL-c and (F) TG.

A

B

D

C

F

E

FPG, fasting plasma glucose; HbA1c, glycated hemoglobin A1c; TC, Total cholesterol; LDL-c, low density lipoprotein cholesterol; HDL-c, high density lipoprotein cholesterol; TG, Triacylglycerol.
